# Quantitative Ubiquitylomic Analysis of the Dynamic Changes and Extensive Modulation of Ubiquitylation in Papaya During the Fruit Ripening Process

**DOI:** 10.3389/fpls.2022.890581

**Published:** 2022-04-25

**Authors:** Yuxing Mo, Bian Jiang, Jingxin Huo, Jiayi Lu, Xiaoyue Zeng, Yan Zhou, Tao Zhang, Min Yang, Yuerong Wei, Kaidong Liu

**Affiliations:** ^1^Life Science and Technology School, Lingnan Normal University, Zhanjiang, China; ^2^Key Laboratory of South Subtropical Fruit Biology and Genetic Resource Utilization (MOA), Guangdong Province Key Laboratory of Tropical and Subtropical Fruit Tree Research, Institute of Fruit Tree Research, Guangdong Academy of Agricultural Sciences, Guangzhou, China

**Keywords:** ubiquitination, fruit ripening, LC-MS/MS, molecular chaperone, ethylene, cell wall

## Abstract

Lysine ubiquitination is a highly conserved post-translational modification with diverse biological functions. However, there is little available information on lysine ubiquitination of non-histone proteins in papaya (*Carica papaya* L.). In total, 3,090 ubiquitination sites on 1,249 proteins with diverse localizations and functions were identified. Five conserved ubiquitinated K motifs were identified. Enrichment analysis showed that many Hsps were differentially ubiquitinated proteins (DUPs), suggesting an essential role of ubiquitination in degradation of molecular chaperone. Furthermore, 12 sugar metabolism-related enzymes were identified as DUPs, including an involvement of ubiquitination in nutrimental changes during the papaya ripening process. The ubiquitination levels of five fruit ripening-related DUPs, including one ethylene-inducible protein, two 1-aminocyclopropane-1-carboxylic acid oxidases, one endochitinase, and one cell wall invertase, were significantly changed during the ripening process. Our study extends the understanding of diverse functions for lysine ubiquitination in regulation of the papaya fruit ripening process.

## Introduction

Papaya (*Carica papaya* L.) is a typical climacteric crop that is widely cultivated in tropical and subtropical areas (Guo et al., 2014). Papaya fruit is rich in a number of nutrient substances (Ming et al., 2008). Ripening is a complex postharvest process consisting of various physiological and biochemical changes, such as sugar conversion, pigment metabolism, cell wall degradation, and flavor biosynthesis (Osorio et al., 2013; Forlani et al., 2019). As a respiratory climacteric fruit, papaya fruit has a short shelf-life, significantly limiting its market sales (Yao et al., 2014). A better understanding of the postharvest changes during the papaya fruit ripening will help to extend its storage period (Gomes et al., 2016).

Fruit ripening is controlled by a series of internal and external factors (Karlova et al., 2014; Hewitt et al., 2020). Enzyme-mediated alterations of the composition and structure of the cell wall is one of the major causes of the ripening process of papaya fruit (Phothiset and Charoenrein, 2014). Several cell-wall modifying enzymes, such as CpPE51, CpXTH12, CpPME1, CpPME2, CpPG5, CpPDS2, CpPDS4, and CpCHY-b, are considered to be involved in fruit ripening (Fu et al., 2016, 2020). Changes in internal hormones also played essential roles in the regulation of the fruit ripening process (Forlani et al., 2019). In papaya fruit, ethylene level is significant at the initiation stage of the ripening process, and a number of ethylene-related transcription factors, such as CpERF9, CpEIL1, and CpEIN3a, are considered to participate in the ripening process (Fu et al., 2016; Zhang T. et al., 2020). Moreover, the effects of environmental factors, such as light and chilling, in the regulation of fruit ripening have were also been reported (Wu et al., 2019; Zou et al., 2020).

Post-translational modification (PTM) is an efficient tactic for aiding the regulation of various cellular processes of plant development and growth (Bond et al., 2011; Shen et al., 2016). Ubiquitination, a deep-studied PTM, plays essential roles in protein degradation and turnover (Wilkinson, 2000). The conjugation of ubiquitin (Ub) to a specific protein is catalyzed by a Ub-activated enzyme (E1), a Ub-conjugated enzyme (E2), and a Ub ligase (E3) (Ye and Rape, 2009). The ε-lysine (K) residues in the target protein function as classic Ub acceptors by forming isopeptide bonds with the carboxyl group of glycine in the C-terminal of Ub (Okumoto et al., 2011). Previous studies revealed the roles of protein ubiquitination in fruit ripening. For examples, a nuclear proteomic analysis revealed the involvement of SlUBC32, an E2 ubiquitin-conjugating enzyme, in tomato fruit ripening (Wang Y. et al., 2014). In strawberries, five Ub-conjugating enzymes differentially accumulated among the four ripening stages (Hu et al., 2018). In apple, a Ub-related gene, *MdFBCP1*, was predominantly expressed in the initial stage of the ripening process, during which ethylene production occurred (Han et al., 2008). Another apple TF, MdbHLH3, which is ubiquitinated by glucose-inhibited Ub E3 ligase MdPUB29, promotes ethylene production during the ripening process (Hu et al., 2019).

To date, very few PTMs have been studied in papaya. Our previous study revealed the lysine crotonylation profiling of papaya fruit, suggesting a potential conserved function of crotonylation in regulation of multiple metabolic pathways (Liu et al., 2018). Despite the importance of ubiquitination in fruit ripening, its role in papaya ripening is largely unknown. Here, the global lysine ubiquitination proteome of papaya fruit was determined using LC-MS/MS with immune-affinity antibody analysis. Our data will expand the understanding of the diverse roles of ubiquitination in regulation of the papaya ripening process.

## Materials and Methods

### Plant Material

Papaya trees (*Carica papaya* L., cv. “Daqing”) were grown in a local commercial plantation near the campus of Lingnan Normal University, Zhanjiang, China. The growth condition was according to our previous published work (Zhang T. et al., 2020). Pre-climacteric red-fleshed papaya fruits (around 120 days post-anthesis) at color break stage with uniform maturity, shape, weight, and free from visual defects were harvested. To remove potential pathogenic bacteria, all fruit samples were washed twice with 0.2% (w/v) hypochlorite for 10 min. Three biological replicates were used for proteomic analysis, each consisting of 15 fruits. Four physiological parameters, including ethylene production, respiration rate, firmness, and titratable acidity, were determined according to our previous study (Zhang T. et al., 2020).

### Physiological Parameter Measurements

Measurements of the physiological parameters of papaya fruit were performed as follows. For the ethylene contents, fruit samples were weighed and put into a 5 L plastic container for 3 h at 25°C. A total of 1 mL sample gases were withdrawn from the container and determined using a Model GC-17A gas chromatograph (Shimadzu Corp., Kyoto, Japan). During the determination, a flame ionization detector and an alumina column were used. the firmness of fruit was determined using a GY-J mini fruit tester (Top Instrument Co., Ltd., Shanghai, China). Then, 500 mg of fruit pulp was crushed with a mortar and pestle to fruit juice. The juice was titrated against 0.1 M NaOH until a faint pink color was obtained at the end point and titratable acidity was expressed as percentage citric acid. For respiration rate determination, papaya fruit samples were weighed and sealed in 2 L plastic containers at 25 °C. The concentration of CO_2_ was measured using an infrared Li-6262 CO_2_/H_2_O gas analyzer (LI-COR, United States).

### Protein Extraction and Trypsin Digestion

About 500-mg fruit samples were first pulverized using a mortar with liquid N_2_. Sample powder was put into 5-mL tube and treated with pre-chilled lysis buffer (150 mM NaCl, 1.0% Nonidet P-40, 50 mM Tris-Cl, pH 7.4) on ice for 5 h. After centrifugation at 15,000 g for 10 min at 4°C, the soluble protein in the supernatant was collected and precipitated with pre-chilled TCA buffer (Trichloroacetic acid, 50% w/v) for 3 h at 4°C. The final precipitate was redissolved in a working solution containing 8 M urea and 100 mM TEAB, pH 8.0, until used.

Concentration of protein solution was analyzed using a 2-D kit (GE Healthcare). The quantified sample solution was alkylated with 20 mM iodoacetamide (Sigma-Aldrich) for 35 min at room temperature in darkness. The protein sample was diluted with 100 mM TEAB and added with trypsin (ChemeGen, Shanghai, China) at a 1:50 mass ratio (trypsin: protein) for the first overnight digestion and at a 1:1,000 mass ratio for the second 4-h digestion. About 100 μg of trypsin digested protein sample was used for the following experiments (Yu et al., 2020).

### Tandem Mass Tag Labeling and HPLC Fractionation

The digested protein samples were desalted using a Strata X C18 SPE column (Phenomenex, United States). Then, protein samples were processed using a six-plex TMT kit (Thermo Fisher Scientific) in 0.5 M TEA solution. The mixture solution was incubated for 2 h at 25°C. After desalted, the sample was vacuum dried.

The sample with tandem mass tag (TMT) label was fractionated by high-pH reverse phase HPLC with an Agilent 300 Extend C18 column (Agilent Technologies Inc., 5-μm particles, 10-mm ID, 250-mm length). Peptide samples were fractionated by acetonitrile buffer at a gradient from 8 to 32% into 60 fractions. Then, all fractions were combined into 18 fractions and vacuum dried.

### Affinity Enrichment and LC-MS/MS Analysis

Tryptic samples were dissolved in NETN buffer, containing 100 mM NaCl, 1 mM EDTA, and 50 mM Tris-HCl, pH 8.0. Then, samples were incubated with pretreated 25D5 type antibody beads (Thermo Fisher Scientific) at 4°C for 12 h under gentle shaking. After enrichment, the antibody beads beading with ubiquitinated peptides were washed twice with the NETN buffer (pH 8.0) and cleaned with ddH_2_O. The peptides binding to the antibody beads were eluted using 0.1% trifluoroacetic acid solution. Before LC-MS/MS uploading, the peptide samples were purified using Millipore C18 ZipTips (Millipore, Darmstadt, Germany) according to its manufacturer’s instructions.

Then, result peptides were dissolved in 0.1% formic acid and loaded onto an EASY-nLC 1000 UPLC system with a reversed-phase analytical column (Thermo Fisher Scientific, 15-cm in length, 75-μm ID). The gradient was set as previously published work (Guo et al., 2017; Hao et al., 2017). The detailed parameters were set as follows: electrospray voltage applied (2.0 kV), *m/z* range for full scan (350–1,800), the resolution of intact peptide detection (70,000), NCE setting (28), resolution of fragment detection (17,500), the automatic gain control (5E4), and the fixed first mass (100 *m/z* ratio). Resulting peptides were subjected to NSI source chromatography followed by MS/MS using a Q Exactive Plus coupled online to the UPLC instrument (Thermo, Shanghai, China).

### MS Data Analysis and Annotation

Maxquant with intergrated Andromeda search engine ver.2.0.3.1^1^ was used for MS/MS data processing (Cox et al., 2011). The MS/MS data were BLASTed against the papaya genome in Phytozome 12 database.^2^ Trypsin/P (NO. A003702, Sangon Biotech, Shanghai, China) was treated as a cleavage enzyme, and less than two missing cleavages were accepted. The mass error for precursor ions was set at 10 ppm, and the mass error for fragment ions was set at 0.02 Da. Two standards, false discovery rate threshold < 1% and the score of peptide ion ≥ 20, were used for peptide identification.

### Classification of Ubiquitinated Protein

For Gene Ontology (GO) annotation, the IDs of all ubiquitinated protein were converted into UniProt IDs. Then, resulting UniProt IDs were mapped onto different GO terms. The GO terms of the remaining proteins were classified by InterProScan software basing on their sequence alignments.

For Kyoto Encyclopedia of Genes and Genomes (KEGG) pathway annotation, all the ubiquitinated proteins were searched against the KEGG database.^3^ Firstly, KEGG online tools KAAS was used to provide KEGG description of ubiquitinated proteins. Then, another KEGG online tools KEGG Mapper was used to provide the sites on the KEGG pathway. Wolfpsort software^4^ was used to predicate the subcellular localizations of ubiquitinated proteins (Yu et al., 2020).

### Functional Enrichment Analysis

A two-tailed Fisher’s exact test was applied to analyze the GO, KEGG, and domain enrichments of the differentially ubiquitinated proteins (DUPs). A multiple hypothesis correction test was performed using the standard false discovery rate control method. The GO, KEGG, and domain categories with an adjusted *P*-value < 0.05 were treated as significant terms. Protein clustering was analyzed using the MeV software with K-means method. All the sequences in the GO-base, KEGG-base and domain-base databases were used as the background.

### QRT-PCR Method

The same fruit samples were used in qRT-PCR experiment. Total RNAs were isolated using a TRlzol reagent (Invitrogen, Shanghai, China) according to its protocol. The quality of RNAs was quantified using a Bioanalyzer 2100 with an RNA integrity parameter > 7.0. the cDNA was synthesized by ReverAid First Strand cDNA Synthesis Kit (Thermo Scientific, Shanghai, China). QRT-PCR was carried out using the SYBR Premix Ex Taq Kit (TaKaRa, Dalian, China) on ABI PRISM 7700 system. A papaya *ACTIN* sequence was used as the internal standard gene to calculate relative fold differences by the values of comparative cycle threshold (2^–ΔΔCt^) (Liu K. et al., 2017). All of the primer sequences are listed in Supplementary Dataset 1.

## Results

### Overview of the Ubiquitome

**FIGURE 1:**
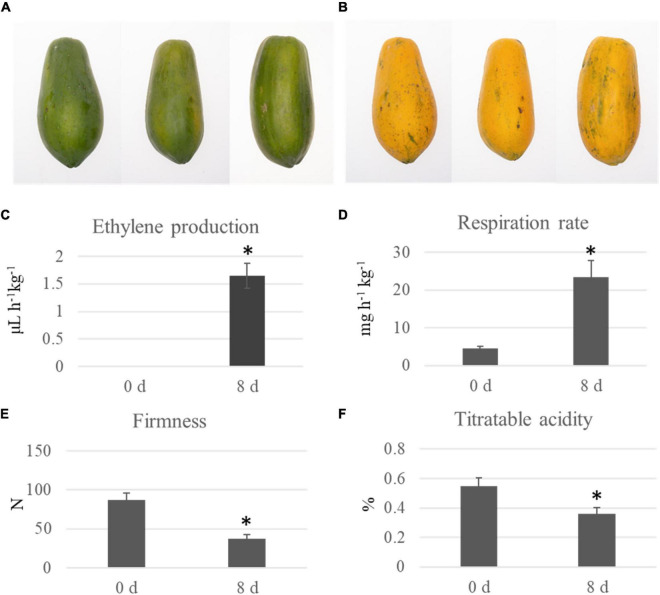
Physiological changes of papaya fruit at time point 0 and 8 d. The picture of papaya fruit at 0 **(A)** and 8 d **(B)**. Four physiological parameters, including ethylene production **(C)**, respiration rate **(D)**, firmness **(E)**, and titratable acidity **(F)**, of papaya fruit at two time points after harvest were analyzed. Significant differences in physiological parameters between time point 0 and 8 d were indicated by “*”.

**FIGURE 2:**
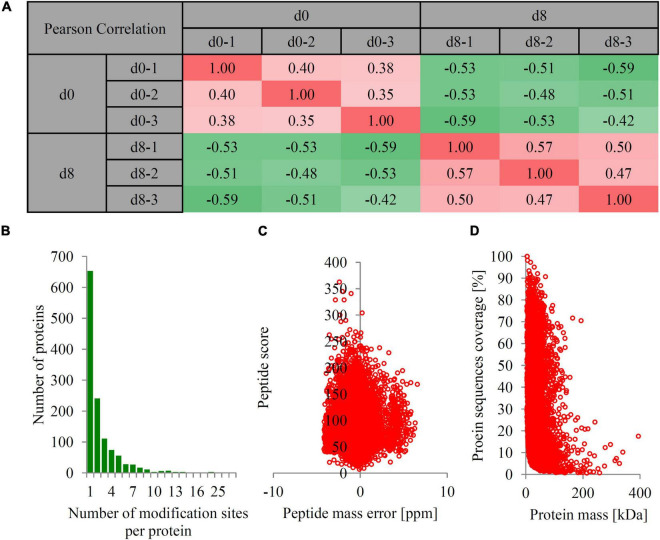
The basic information of LC-MS/MS data. **(A)** Pearson’s correlation coefficient analysis of the data from different sample groups. The red color indicated positive correlation and green color indicated negative correlation. **(B)** The number of modification sites per protein. **(C)** The peptide mass error (ppm). **(D)** The protein mass [kDa]. The mass error threshold for peptide ions was set at 10 ppm, and the mass error threshold for protein was set at 0.02 Da.

Two groups of papaya fruit at time points of 0 and 8 d after harvest were collected for ubiquitomic analysis (Figures 1A,B). Four important morphological and physiological parameters, including ethylene production, respiration rate, firmness, and titratable acidity, were analyzed to check the sampling time points is appropriate. At time point 8 d, the ethylene production was significantly up-regulated from 0 to 1.65 μL h^–1^kg^–1^, the respiration rate increased from 4.53 to 23.45 mg h^–1^kg^–1^, the fruit firmness down-regulated from 87.3 to 37.3 N, and the titratable acidity decreased from 0.55 to 0.36% (Figures 1C–F). Our data showed that fruit collected at time point 8 d can represent the mature fruit. Quantitative ubiquitomic analysis was performed to uncover the ubiquitomic changes of papaya fruit during ripening process. Pearson’s correlation analysis showed that the sample groups were greatly reproducible (Figure 2A). Two quality parameters, length distribution and mass error, were analyzed to verify the MS data. Most peptides varied from 7 to 19 amino acids in size (Figure 2B) and mass errors were lower than 5 ppm (Figures 2C,D), suggesting that data quality meet the standard.

In total, 1,249 proteins with 3,090 ubiquitination sites were identified, of which 1,142 proteins with 2,731 ubiquitination sites were quantified. Among them, about half of the ubiquitinated proteins (653 proteins) contain only one ubiquitination site and 596 ubiquitinated proteins contain more than two ubiquitination sites. Three proteins contain more than 25 ubiquitination sites, including heat shock cognate 80 (30 sites), BAG family molecular chaperone regulator 7 (26 sites), and Patellin-5 -like protein (25 sites). The detailed information for each ubiquitination site, including position, protein description, modified sequence, mass error, and MS/MS count, are listed in Supplementary Dataset 2.

**FIGURE 3:**
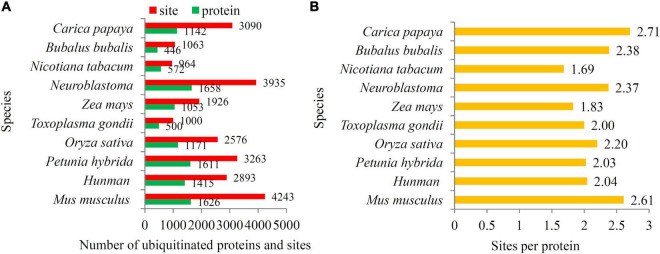
Analysis of the differences between papaya and other published organisms. **(A)** The number of ubiquitinated proteins and ubiquitination sites in papaya and other published organisms. Red bars indicated the number of ubiquitinated proteins and green bars indicated the number of ubiquitinated sites. **(B)** The number of ubiquitination sites per protein in papaya and other published organisms, including Bubalus bubalis, Nicotiana tabacum, Neuroblastoma, Zea mays, Toxoplasma gondii, Oryza sativa, Petunia hybrida, Human, and Mus musculus.

As an important PTM, ubiquitylomics of many other organisms have been published (Jiang et al., 2018; Li et al., 2018; Wang et al., 2019; Cai et al., 2020; Zhang P. F. et al., 2020). Our study identified 3,090 ubiquitination sites in papaya, which is more than found in *Toxoplasma gondii* (1,000 sites), *Zea mays* (1,926 sites), *Nicotiana tabacum* (964 sites), *Oryza sativa* (2,576 sites), and *Bubalus bubalis* (1,063 sites), and is less than in *Mus musculus* (4,243 sites), *Neuroblastoma* (3,935 sites), and *Petunia hybrida* (3,263 sites) (Figure 3A). In addition, there are 2.71 ubiquitination sites per protein in papaya. The densities of ubiquitination site of most reported organisms are > 2.0, except for *Zea mays* (1.8 sites per protein) and *Nicotiana tabacum* (1.7 sites per protein) (Figure 3B).

### Functional Classification of All Ubiquitinated Proteins

**FIGURE 4:**
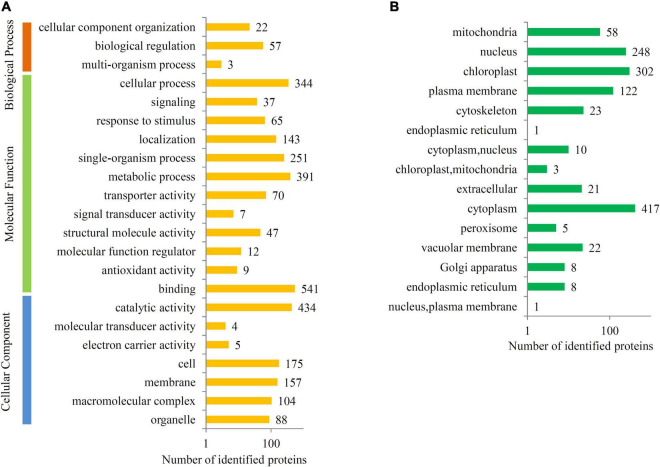
Functional classification of the ubiquitinated proteins. **(A)** GO analysis of all identified ubiquitinated proteins. All the proteins were groups into three major categories, such as biological process, molecular function, and cellular component. **(B)** Subcellular location analysis of all identified ubiquitinated proteins. The number of proteins present in each GO class or subcellular location was provided to each bar.

To predict the biological functions of the ubiquitinated proteins, GO, KEGG, and subcellular localization were examined. Most of the ubiquitinated proteins were assigned to at least one GO term. The top “Biological Process” GO terms were “cellular component organization” (22 proteins) and “biological regulation” (57 proteins), and the top “Molecular Function” GO terms were “binding” (541 proteins), “metabolic process” (391 proteins), and “cellular process” (344 proteins). The top “Cellular Component” GO terms were “catalytic activity” (434 proteins), “cell” (175 proteins), and “membrane” (157 proteins) (Figure 4A).

For subcellular locations, most ubiquitinated proteins in papaya fruit were classed into 15 major categories, including 417 cytoplasm proteins, 302 chloroplast proteins, 248 nucleus proteins, and 122 plasma membrane proteins (Figure 4B).

### Differentially Ubiquitinated Proteins Responsive to the Ripening Process

**FIGURE 5:**
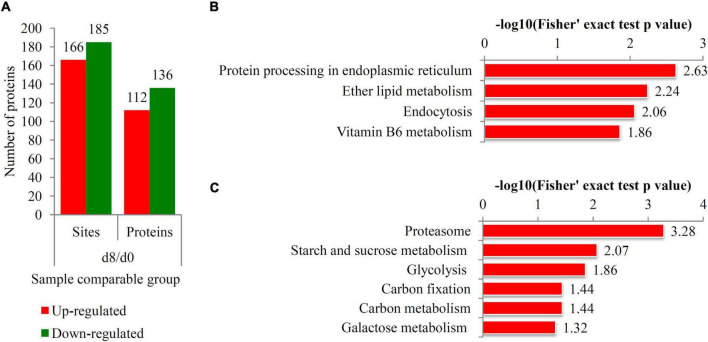
Identification of differentially ubiquitinated proteins responsive to ripening process. **(A)** The number of up- and down-regulated ubiquitinated proteins during the fruit ripening process. Red bars indicated the up-regulated proteins and green bars indicated the down-regulated proteins. **(B)** KEGG analysis of the up-regulated ubiquitinated proteins. **(C)** KEGG analysis of the down-regulated ubiquitinated proteins. The negative logarithm of Fisher’s exact test *P*-value was showed in × axes.

The differential ubiquitination levels of each protein during the ripening process are listed in Supplementary Dataset 3. During the ripening process, 112 proteins with 166 ubiquitination sites were up-regulated and 136 proteins with 185 ubiquitination sites were down-regulated (Figure 5A). The top-five significantly induced ubiquitination sites were 26K in ethylene-inducible protein (9.1-fold), 71K and 147K in 17.3 kDa class I heat shock protein-like (8.9- and 7.7-fold, respectively), 29K in class I heat shock protein-like protein (7.9-fold), and 457K in heat shock 70 kDa protein (7.6-fold). The significantly down-regulated ubiquitination sites were 1112K in Ub carboxyl-terminal hydrolase 12 (0.074-fold), 354K in fructose-bisphosphate aldolase (0.079-fold), and 10K in BRCA1-A complex subunit BRE (0.085-fold) (Supplementary Dataset 3).

Most of the DUPs were assigned to different KEGG terms. For the up-regulated ubiquitinated proteins, the most significantly enriched KEGG terms were “Protein processing endoplasmic reticulum” (*P* = 2.63), “Ether lipid metabolism” (*P* = 2.24), “Endocytosis” (*P* = 2.06), and “Vitamin B6 metabolism” (*P* = 1.86) (Figure 5B). For the down-regulated ubiquitinated proteins, the most significantly enriched KEGG terms were “Proteasome” (*P* = 3.28), “Starch and sucrose metabolism” (*P* = 2.07), “Glycolysis” (*P* = 1.86), “Carbon fixation” (*P* = 1.44), “Carbon metabolism” (*P* = 1.44), and “Galactose metabolism” (*P* = 1.32) (Figure 5C).

Ubiquitinated proteins in papaya were mostly highly enriched in α-crystallin/Hsp20 (*P* = 2.48), C2 (*P* = 2.47) and Hsp20like chaperone (*P* = 2.47) domains (Supplementary Figure 1).

### Differentially Ubiquitinated HSPs and Other Chaperone Proteins

Protein domain enrichment analysis identified several Hsp domains as DUPs, such as Hsp20 (*P* = 2.48), Hsp20-like (*P* = 2.47), and Hsp90 (*P* = 2.04). The roles of Hsp proteins in environmental response and organ development have been deeply investigated (Shan et al., 2019). In our study, a number of Hsp proteins were identified as DUPs, including 17.3-, 70-, 80-, 17.5-, and 20-kDa types (Supplementary Dataset 4). The ubiquitination levels of these identified Hsps significantly changed during the fruit ripening process. Interestingly, most ubiquitination sites in Hsps were up-regulated in the ripening process. For example, a predicted 17.3-kDa type Hsp (Unigene0022810) contained four up-regulated ubiquitination sites, and a predicted Hsp80 (Unigene0036152) contained seven up-regulated ubiquitination sites.

In addition to Hsps, several chaperone proteins were also identified as DUPs. A BAG family molecular chaperone regulator 7 (Unigene0002491) containing four down-regulated ubiquitination sites, a DnaJ protein (Unigene0003516) with one down-regulated ubiquitination site, and a DnaJ 10-like protein (Unigene0028971) with one up-regulated ubiquitination site were identified (Supplementary Dataset 4).

### Analysis of Primary Metabolism-Related Differentially Ubiquitinated Proteins

During the ripening process, dynamic changes in the nutrient components are observed in various fruits. In this study, 12 sugar metabolism-related enzymes with 15 ubiquitination sites were identified. In detail, a hexokinase (Unigene001480) with one up- and two down-regulated sites, a fructose-bisphosphate aldolase (Unigene0031374) with one up- and two down-regulated sites, a fructokinase (Unigene0016829) with two down-regulated sites, a sucrose synthase 4 isoform (Unigene0020356) with one up- and one down-regulated site, two glyceraldehyde-3-phosphate dehydrogenase isoforms (Unigene0027871 and Unigene0028637) with one site each, an aconitase 3 (Unigene0029018) with one down-regulated site, and a malate dehydrogenase (Unigene0029490) with one up- and one down-regulated site, were identified as DUPs (Supplementary Dataset 5).

### Analysis of Fruit Ripening-Related Differentially Ubiquitinated Proteins

Previous studies revealed many fruit ripening-related factors in different plants. In this study, five fruit ripening-related DUPs, including one ethylene-inducible protein (Unigene0021524), two ACC oxidases (Unigene0002414 and Unigene0037522), one endochitinase (Unigene0036632), and one cell wall invertase (Unigene0033279), were identified (Supplementary Dataset 5). Interestingly, three ubiquitination sites in the ethylene-inducible protein were up-regulated during the ripening process. Both ACC oxidases contained two ubiquitination sites, with their ubiquitination levels significantly up-regulated during the ripening process. Our data showed that both endochitinase and cell wall invertase contained one down-regulated ubiquitination site each (Supplementary Dataset 6).

### Expression Verification of Several Key Genes by qRT-PCR

To verify the changes in expression levels of several DUP encoding genes, qRT-PCR was performed. Six primary metabolism and fruit ripening-related DUP encoding genes, including hexokinase (Unigene001480), aconitase 3 (Unigene0029018), malate dehydrogenase (Unigene0029490), ACC oxidases (Unigene0002414), cell wall invertase (Unigene0033279), and ethylene-inducible protein (Unigene0021524), were selected. Our data showed that hexokinase, malate dehydrogenase, ACC oxidases, cell wall invertase, and ethylene-inducible protein encoding genes were significantly up-regulated during the ripening process (Supplementary Figure 2). Both of transcriptional regulation and PTM occurred during the fruit ripening process of papaya.

## Discussion

A range of evidence proved that ubiquitinated proteins play essential roles in different biological processes, such as cellular protein degradation, apoptosis, and signal transduction (Jiang et al., 2018; Cai et al., 2020). Recent studies have revealed that protein ubiquitination is involved in the fruit ripening process (Wei et al., 2020). Investigation of the lysine ubiquitination proteome of papaya fruit will broaden our understanding the roles of ubiquitinated proteins in the fruit ripening process.

Many studies on ubiquitination proteomes have been recently performed in different species, including mammals, microbes, and plants (Silmon de Monerri et al., 2015; Guo et al., 2017; Jiang et al., 2018; Li et al., 2018; Meng et al., 2018; Wang et al., 2019; Cai et al., 2020; Zhan et al., 2020; Zhang P. F. et al., 2020). The number of ubiquitination sites in papaya is more than for most reported organisms, suggesting a high degree of ubiquitination in papaya. In reported plants, high ubiquitination levels were detected in papaya fruit and *petunia* flower, and low ubiquitination levels in rice seeds, maize seedlings, and tobacco leaves (Figure 4A). In addition to ubiquitination sites, the numbers of ubiquitinated protein also significantly differed among the reported organisms (Figure 4B). Among the published ubiquitylomic data sets, papaya possessed the largest number ubiquitination sites in each protein (2.7 sites per protein), which exceeded the number of ubiquitination sites in other plants, such as tobacco (1.69 sites per protein) and maize (1.83 sites per protein). A higher ubiquitination level in papaya fruit indicated an important role of ubiquitinated proteins in fruit ripening.

Previous study revealed that Hsp proteins were involved in the regulation of the fruit ripening process. For example, the expression level of *SlHSP17.7A* and *SlHSP17.7B* were significantly changed in tomato ripening mutants compared with wild type (Upadhyay et al., 2020). During the early development stage, several Hsp genes showed differential expression levels in litchi (Liu W. et al., 2017). Domain enrichment analysis showed that many of the ubiquitination levels of proteins containing the Hsp domain were significantly changed during the fruit ripening process. In grape embryogenic callus, down-regulated Hsp70 protein was involved in the Ub-associated protein-degradation pathway (Zhao et al., 2011). Interestingly, several molecular chaperones contained more than 20 ubiquitination sites, suggesting an essential role of ubiquitination in degradation of molecular chaperones. In plants, Hsp80 and Hsp83 are involved stress responses. In tomato plant, Hsp80 is an important component of the melatonin-induced stress tolerance (Gong et al., 2017). In *Arabidopsis*, the expression level of Hsp83 is rapidly enhanced by elevated temperature (Conner et al., 1990). In papaya, Hsp80 (Unigene0036152) contains ubiquitination 30 sites, and Hsp83 (Unigene0039978) contains ubiquitination 21 sites. Our data suggested that ubiquitination might be involved in stress responses by regulating the stability of Hsp family proteins.

Accumulation of nutrient components, such as amino acids and sugars, contributes to improving the nutritional quality of papaya fruit (Santana et al., 2019). Glycolysis was reported to be involved in the ripening process of climacteric fruits (Ball et al., 1991). However, the proteins involved in sugar metabolism of papaya fruit are largely unknown. In our study, proteomic analysis identified several sugar metabolism-related enzymes. In grape, hexokinase and sucrose synthase played roles in sugar sensing and signaling (Wang X. Q. et al., 2014). In our study, both of hexokinase and sucrose synthase contained one up- and one down-regulated ubiquitination sites, suggesting a role of ubiquitination in energy metabolism during the fruit ripening process. Furthermore, induced malate content has the potential to improve the taste of early ripening apple cultivars (Onik et al., 2019). Cytosolic malate dehydrogenase played an essential role in malate synthesis throughout fruit development, especially in the ripening apple fruit (Yao et al., 2011). Our data showed that malate dehydrogenase in papaya was DUP, indicating that ubiquitination might be involved in the regulation of malate content during the ripening process. Fructose-1, 6-bisphosphate aldolase is important enzyme in the Calvin-Benson cycle. In tomato, fructose-1, 6-bisphosphate aldolase showed an essential role in regulating growth and chilling tolerance of tomato seedlings (Cai et al., 2018). In papaya, the ubiquitination level of fructose-bisphosphate aldolase was significantly decreased during the fruit ripening process, suggesting a role of ubiquitination in chilling tolerance of papaya fruit.

The plant hormone ethylene has been well studied to concerning its role in fruit ripening (Kumar et al., 2014). At the initial stage of the ripening process, a peak of endogenous ethylene was detected in papaya fruit (Oliveira et al., 2015), and application of exogenous ethylene can quicken the ripening process of papaya fruit (Shen et al., 2017). Recently, many ethylene biosynthesis pathway-related genes have been isolated from papaya (Li et al., 2013; Shen et al., 2017). In papaya fruit, 1-aminocyclopropane-1-carboxylic acid (ACC) oxidase is an important enzyme involved in triggering the system II ethylene biosynthesis during ripening (Fabi and do Prado, 2019). Many years ago, a genomic clone of ACC oxidase from papaya was identified (Lopez-Gomez et al., 2004). However, studies on the PTM of ethylene biosynthesis-related proteins are very rare. In the present study, we identified two homologous proteins of ACC oxidase, and two ubiquitination sites on each ACC oxidase were detected. In model plants, XBAT32, RING-type E3 Ub ligase, ubiquitinates two ethylene biosynthesis enzymes, ACS4 (type-II isoform) and ACS7 (type-III isoform) (Lyzenga et al., 2012). Interestingly, all ubiquitination sites were significantly up-regulated during the ripening process. High ubiquitination level indicated activation of an Ub-mediated protein degradation process of ACC oxidase. Furthermore, a number of ethylene-inducible proteins, such as TCTP and NEIP2 in tobacco, and SAUR76 and ARGOS in Arabidopsis, were identified as inhibitors of ethylene response but activators of plant growth (Tao et al., 2015). In papaya, the ubiquitination level of ethylene-inducible protein significantly changed during the ripening process, indicating a role of ubiquitination in ethylene-mediated fruit ripening.

Fruit softening and ripening are due to the dissolution of the cell-wall middle layer (Paniagua et al., 2017). Cell wall metabolism-related enzymes play essential roles in regulation of physiological and biochemical phenomena associated with fruit ripening (Cardenas-Coronel et al., 2016). In our study, both of endochitinase and cell wall invertase contained one reduced ubiquitination site, indicating a function of ubiquitination in regulation of cell wall degradation. Ethylene, together with other hormones, modulated the ripening process by regulating cell wall degradation-related enzymes (Sharon et al., 1993; Guo et al., 2014). In papaya fruit, the ubiquitination levels of ethylene-related proteins and cell wall-related proteins showed opposite responses to ripening process. This suggested that ethylene might participate in the regulation of fruit ripening by accelerating cell wall degradation. In plants, endochitinase and cell wall invertase, are a kind of pathogenesis-induced proteins, play an important role in defense mechanism and stress tolerance (Hayes et al., 2010; Ben-Amar et al., 2022). Papaya is easily infected with a variety of pathogen-caused diseases. Ubiquitination might be involved in pathogen defense by altering cell wall structure.

Our data provided a comprehensive global lysine ubiquitination proteome of papaya fruit. The basic information of ubiquitinated proteins could be used as raw resources for functional validation of ubiquitinated proteins during the fruit ripening process.

## Data Availability Statement

The datasets presented in this study can be found in online repositories. The names of the repository/repositories and accession number(s) can be found in the article/Supplementary Material.

## Author Contributions

KL conceived, designed the experiments, and provided essential ideas. YM, BJ, JH, JL, YZ, and MY performed the experiments. JL, TZ, and YW analyzed the data. YM and KL wrote and revised the manuscript. All authors reviewed the manuscript.

## Conflict of Interest

The authors declare that the research was conducted in the absence of any commercial or financial relationships that could be construed as a potential conflict of interest.

## Publisher’s Note

All claims expressed in this article are solely those of the authors and do not necessarily represent those of their affiliated organizations, or those of the publisher, the editors and the reviewers. Any product that may be evaluated in this article, or claim that may be made by its manufacturer, is not guaranteed or endorsed by the publisher.

## Footnotes

1
https://maxquant.net/maxquant/

2
https://phytozome-next.jgi.doe.gov/

3
http://www.genome.jp/kegg/

4
https://www.genscript.com/wolf-psort.html
