# Supplementary figures and images for: Quantitative Ubiquitylomic Analysis of the Dynamic Changes and Extensive Modulation of Ubiquitylation in Papaya During the Fruit Ripening Process
Source: Front Plant Sci. 2022 Apr 25;13:890581. doi: 10.3389/fpls.2022.890581 (PMC9082147; doi:10.3389/fpls.2022.890581)

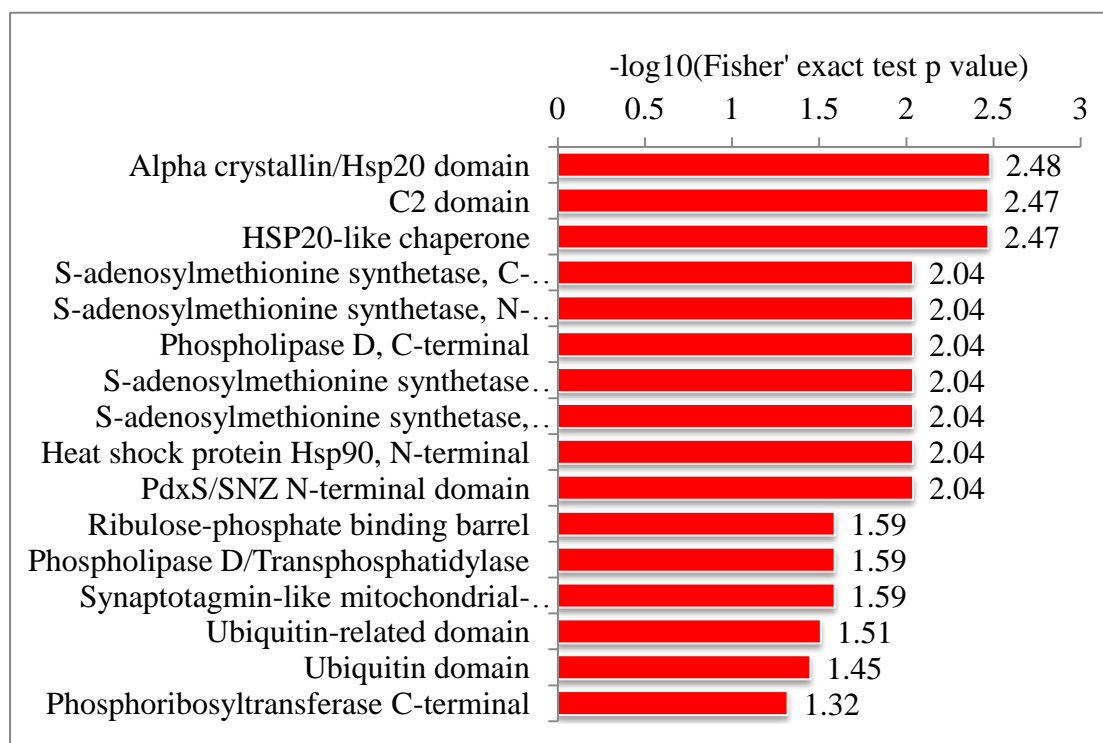

Figure S1 Protein domain enrichment analysis of the ubiquitinated proteins in papaya.

Supplement: Supplementary file 6 [file Data_Sheet_1.PDF]
