# Supplementary material for: Quantitative Ubiquitylomic Analysis of the Dynamic Changes and Extensive Modulation of Ubiquitylation in Papaya During the Fruit Ripening Process
Source: Front Plant Sci. 2022 Apr 25;13:890581. doi: 10.3389/fpls.2022.890581 (PMC9082147; doi:10.3389/fpls.2022.890581)
Supplement: Supplementary file 7 [file Data_Sheet_2.PDF]

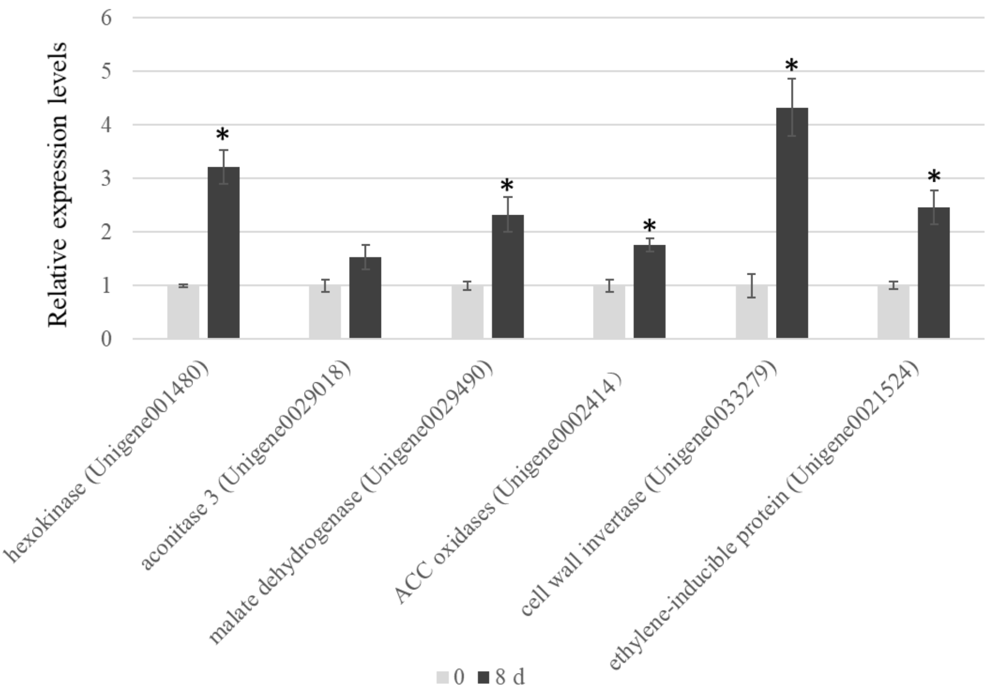

Supplementary Figure S2 Expression verification of several key genes by qRT-PCR. “*” indicated significant differences between 0 and 8d sample groups.
